# Disseminated Central Nervous System Histoplasmosis: A Case Report

**DOI:** 10.7759/cureus.4238

**Published:** 2019-03-12

**Authors:** Hector H Gonzalez, Manas Rane, Anthony Cioci, Sarah Goodman, Patricio S Espinosa

**Affiliations:** 1 Internal Medicine, Florida Atlantic University Charles E. Schmidt College of Medicine, Boca Raton, USA; 2 Neurology, Marcus Neuroscience Institute at Boca Raton Regional Hospital, Boca Raton, USA

**Keywords:** disseminated histoplasmosis, central nervous system, ring-enhancing lesion

## Abstract

Central nervous system (CNS) histoplasmosis is a rare manifestation of disease, often misdiagnosed due to the wide spectrum of neurological presentation. We present a rare case of CNS histoplasmosis in a 62-year-old male with untreated myeloproliferative disease who presented with altered mental status. This case emphasizes the clinical presentation and diagnostic difficulty in a patient with CNS histoplasmosis. We also highlight the importance of implementing a multidisciplinary approach in the medical management of disseminated histoplasmosis with CNS involvement.

## Introduction

Histoplasmosis is a common and frequently self-limiting infection caused by the dimorphic fungus *Histoplasma capsulatum*. Endemic to the Ohio and Mississippi River valleys of the United States, infection with *Histoplasma capsulatum* occurs following inhalation of aerosolized fungal spores [1-2]. According to the Centers for Disease Control (CDC), as many as 60-90% of people living in these endemic areas have been exposed to *Histoplasma* during their lifetime, with as many as 500,000 new infections yearly [1,3,4]. In the majority of cases, illness in the immunocompetent patient is subclinical and frequently goes unrecognized [4]. When clinically notable illness occurs, it typically confined to the respiratory system. Less commonly, infection with *Histoplasma* may result in hematogenous spread of the fungus to distant organs, resulting in disseminated histoplasmosis. While the risk for developing disseminated disease is rare in the immunocompetent patient, those with immunodeficiency are at an increased risk compared to the general population and often experience poorer outcomes [4-6].

Involvement of the central nervous system (CNS) has been reported to occur in 5-10% of disseminated histoplasmosis cases [7]. In those with CNS involvement, clinical features typically include headache, altered mental status (AMS), confusion, or focal deficits, in addition to the signs of disseminated disease such as fever, weight loss, or pulmonary symptoms [4,6,8-10]. When CNS histoplasmosis is suspected, diagnosis can be made by confirming the presence of *Histoplasma* antigen and anti-*Histoplasma* antibodies in either the CNS or serum, with newer assays showing improved sensitivity in recent years. Nonetheless, the diagnosis of CNS histoplasmosis is frequently missed due to misdiagnosis or failure to consider histoplasmosis in areas where it is not endemic [6]. Here we present a case of CNS histoplasmosis in a patient with myeloproliferative disorder who presented with rapid change of mental status.

## Case presentation

A 62-year-old male from Tennessee with a past medical history of janus kinase 2 (JAK2) positive essential thrombocytosis diagnosed in 2014, hypertension, hyperlipidemia, and major depressive disorder presented to the emergency department (ED) with acute encephalopathy. Due to initial encephalopathy, information regarding medical history was obtained by review of previous hospital documentation obtained from his hometown. The patient was previously taking anagrelide 0.5 mg twice a day (BID) then hydroxyurea 500 mg BID for myeloproliferative disorder, however, he discontinued the medications in 2015 due to depression.

His medical history is notable for a hospitalization four months ago where he presented with fatigue, weight loss, and cough, and was found to have an enlarged spleen. During that time, a computerized tomography (CT) scan of the chest, abdomen and pelvis was performed which noted 4 x 4 x 2.3 cm mass in the right adrenal gland, and splenomegaly. Further workup at that time revealed multiple brain lesions on brain imaging, with an unclear source. The patient also had a lumbar puncture (LP) with normal cerebrospinal fluid (CSF) results, negative human immunodeficiency virus (HIV), negative acid-fast stain and toxoplasmosis. Bone marrow biopsy in the past revealed findings consistent with a myeloproliferative disorder. The patient was scheduled to undergo adrenal biopsy, however, he declined this and decided to leave the hospital. Additionally, the patient saw a neurologist one month ago for persistent neck pain for the past seven months, associated with numbness and tingling in his right hand, which had subsequently progressed to his right forearm and upper arm. Magnetic resonance imaging (MRI) of the brain showed numerous supratentorial and infratentorial ring enhancing lesions. The differential diagnosis at that time included metastatic disease, and atypical infection such as toxoplasmosis. A lumbar puncture was done, however, official results were not available except for the cytology which was showing no evidence of malignancy. The week prior to this hospital admission, the patient had a transient episode of difficulty speaking. He called his doctor and was advised to go to the ED for evaluation, however, he declined to do so.

**Figure 1:**
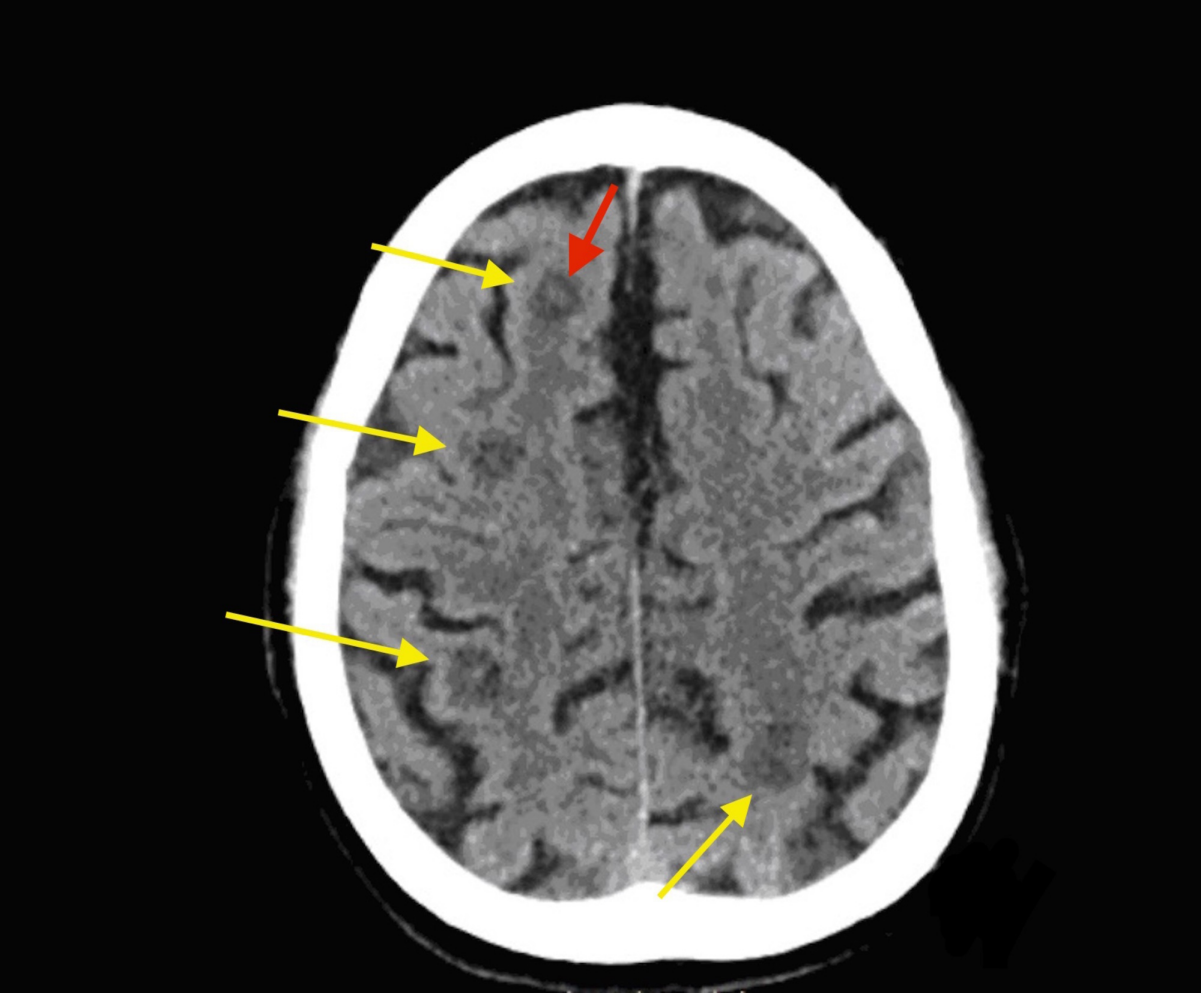
Computed tomography (CT) scan of the head showing hypointense lesions in the fronto-parietal region (yellow arrows). There is hyperintense signal in the center of this lesion consistent with hemorrhage (red arrow).

The patient presented to our hospital this time with altered mental status for the past 24 hours. He was unable to give any history at the time of presentation, although a friend at bedside was able to recall the patient complaining of fevers, weakness, and a headache the day before. Upon arrival to the ED, he was tachycardic, febrile, and saturating well on room air. Initial labs revealed an elevated creatinine of 1.5 mg/dL and an elevated lactic acid of 2.7 mmol/L. The patient was started on broad spectrum antibiotics with cefepime and levaquin. CT of the brain without contrast showed multiple hypointense lesions with a hemorrhagic component (Figure 1).

**Figure 2:**
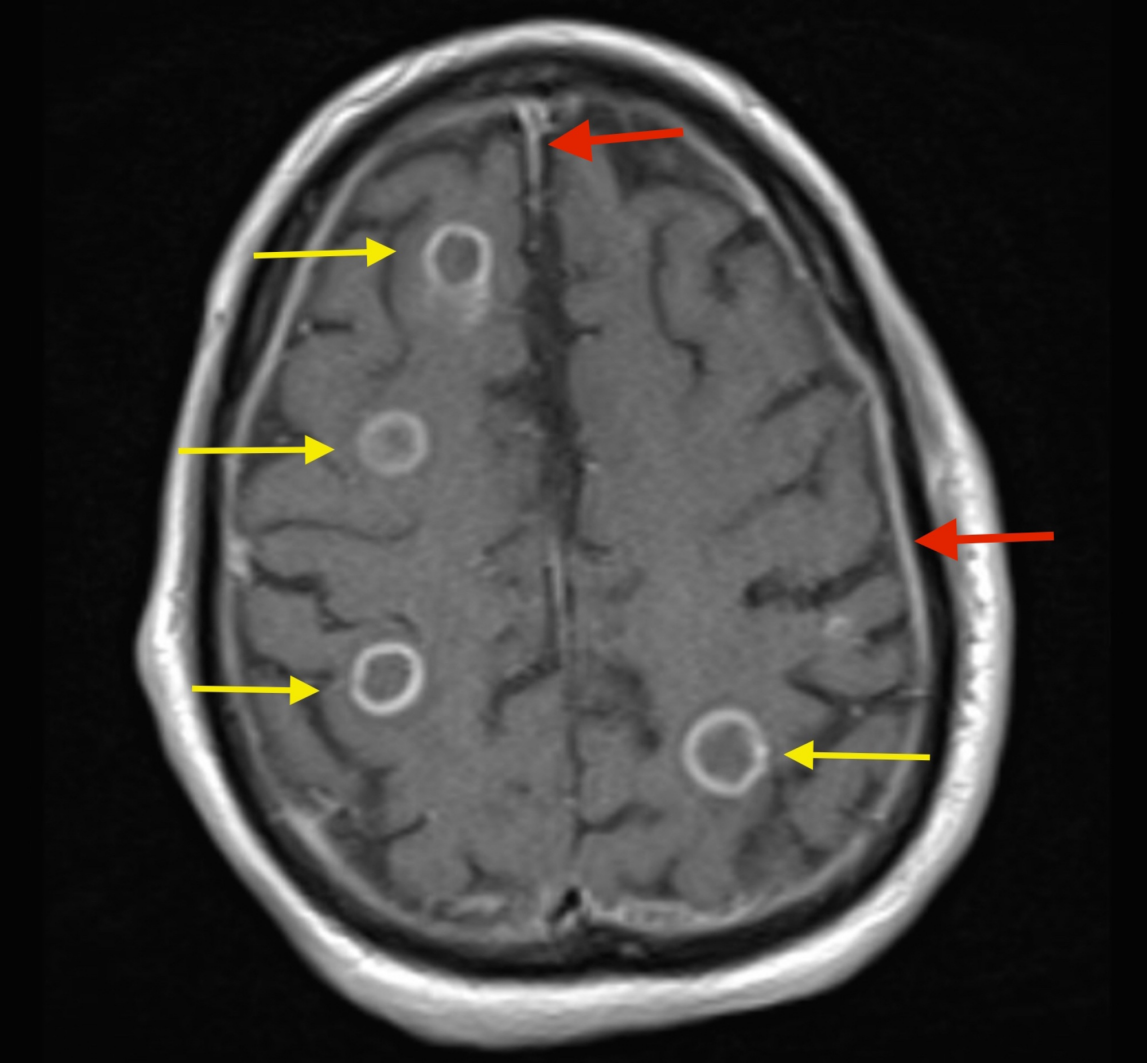
Magnetic resonance imaging (MRI) of the brain with contrast showing numerous ring enhancing lesions throughout brain parenchyma (yellow arrows) with meningeal enhancement (red arrows).

**Figure 3:**
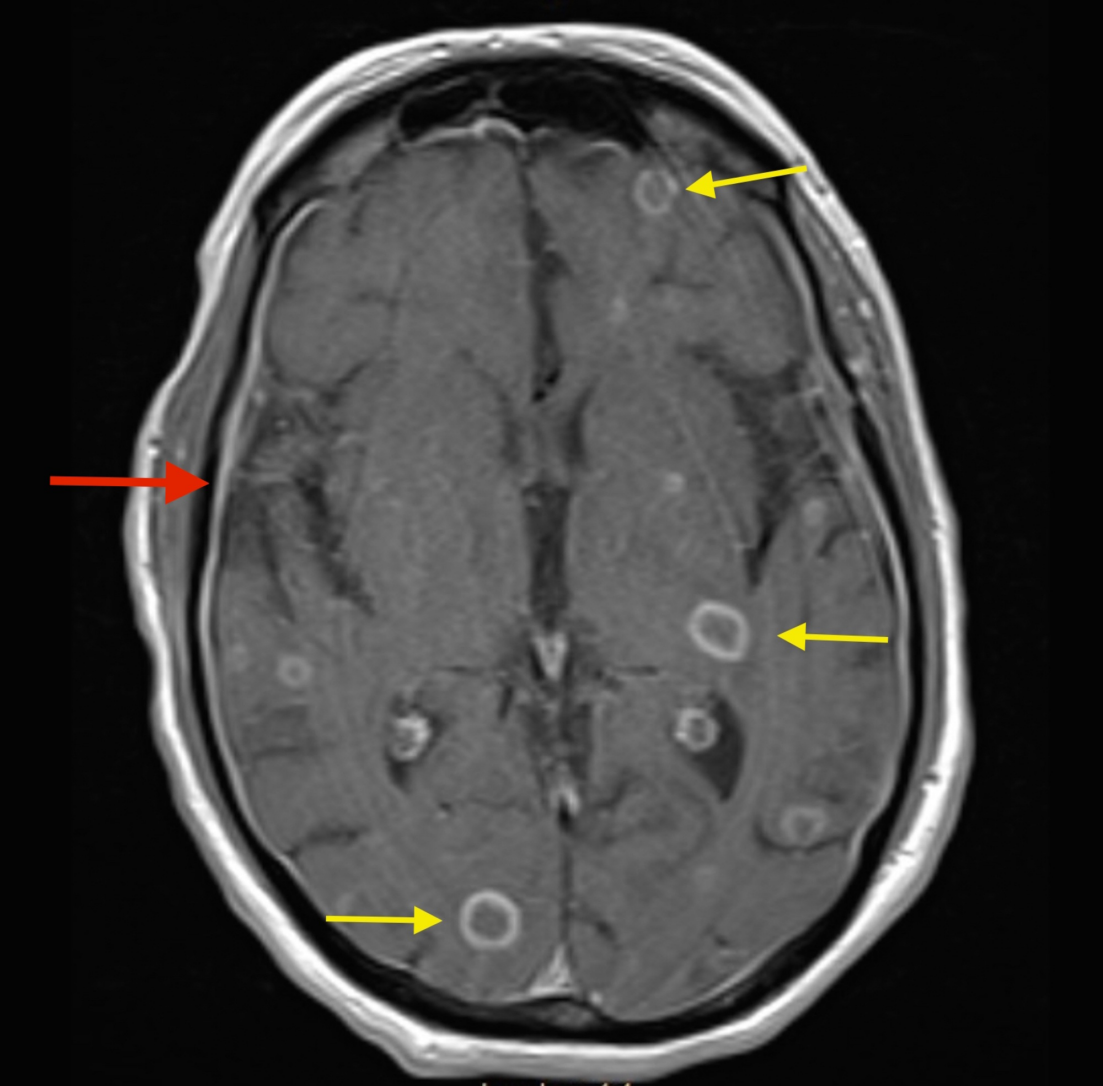
Magnetic resonance imaging (MRI) of the brain with contrast depicting meningeal enhancement (red arrow) with ring-enhancing lesions involving multiple lobes (yellow arrows).

An MRI of the brain with contrast was done which revealed numerous masses throughout the brain parenchyma involving all lobes (Figures 2-3).

**Figure 4:**
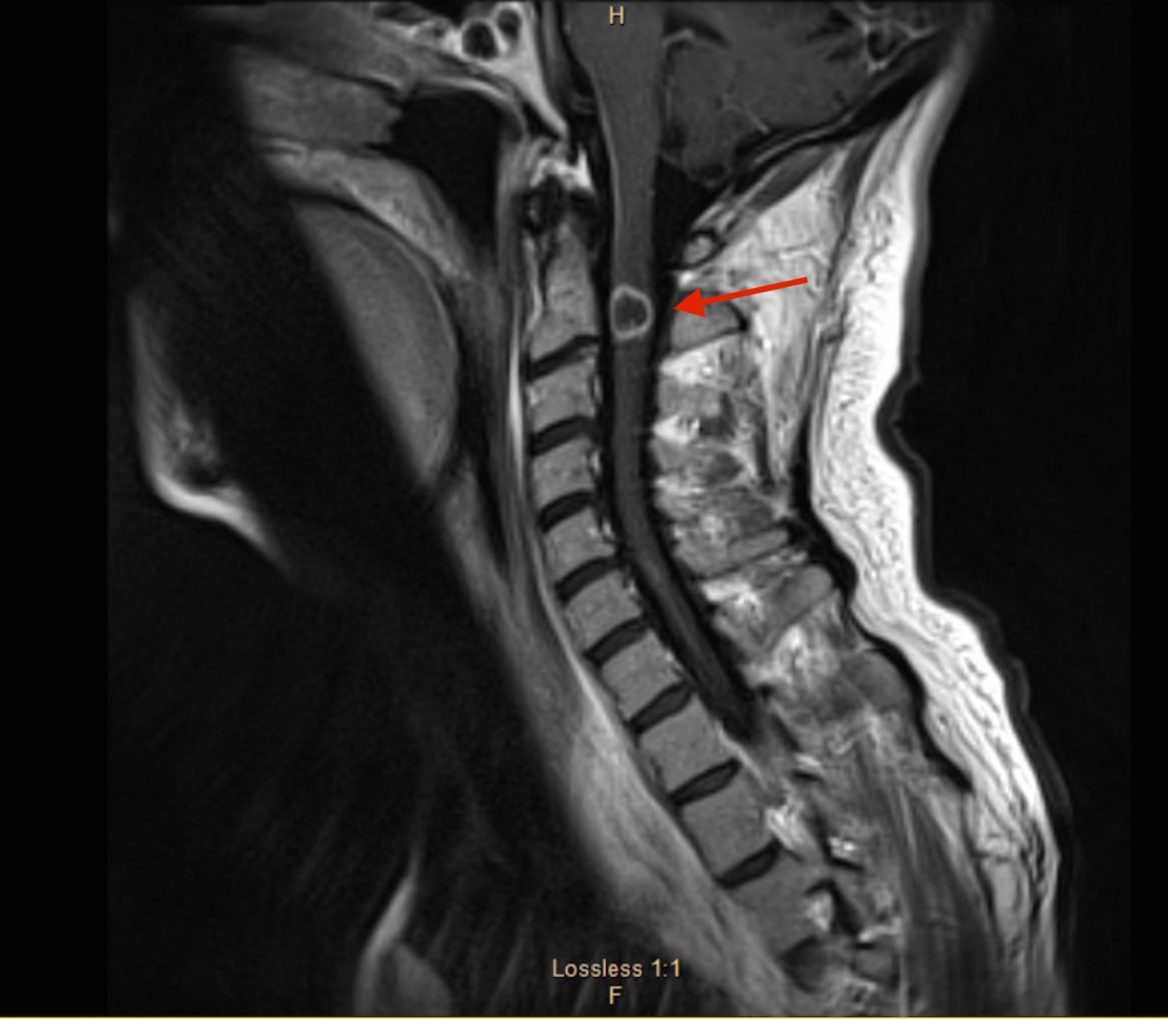
Magnetic resonance imaging (MRI) of the cervical spine showing solitary ring enhancing lesion at C2 (red arrow).

**Figure 5:**
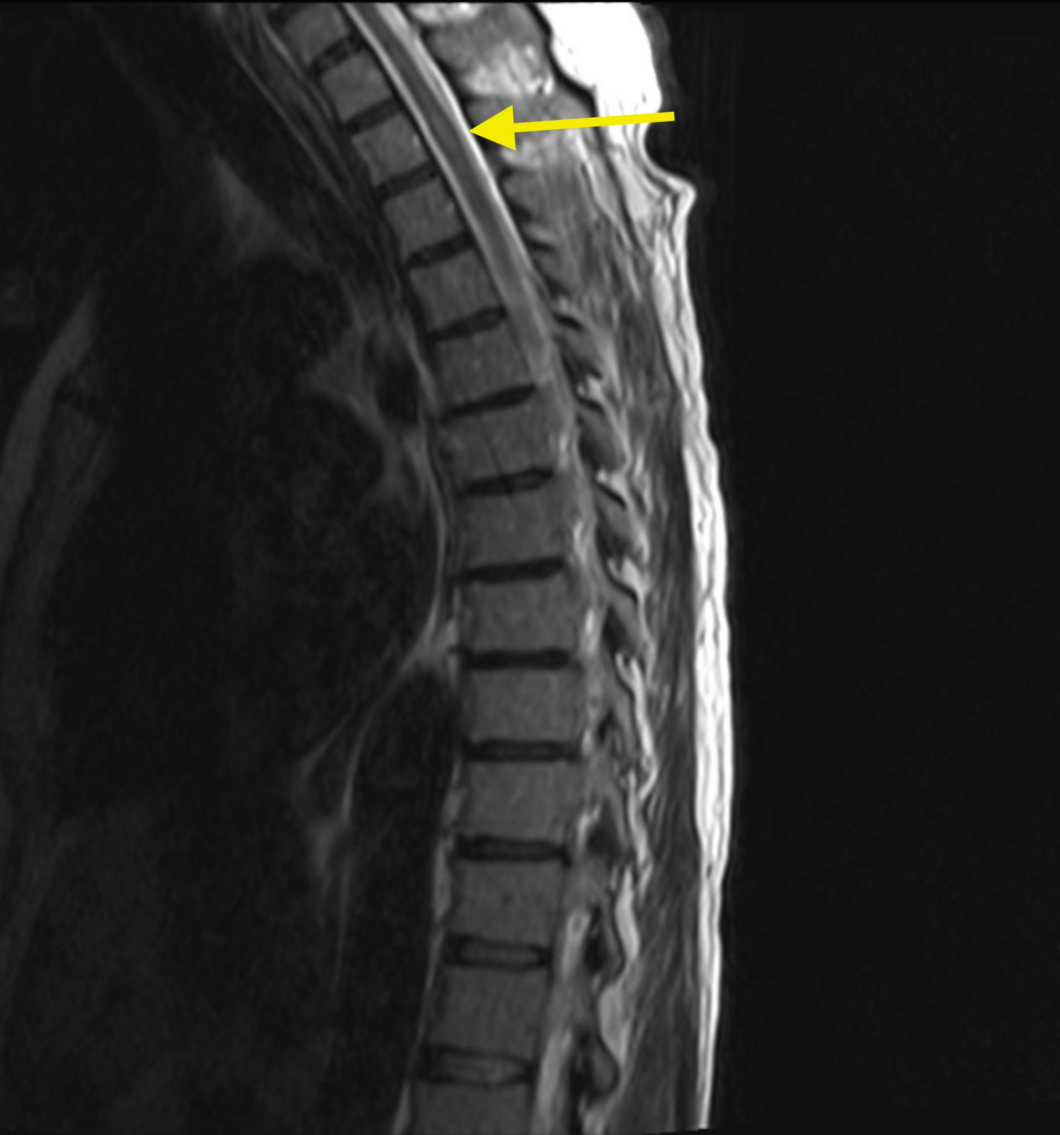
Magnetic resonance imaging (MRI) of the thoracic spine revealing hyperintense lesion at T2 level (yellow arrow).

**Figure 6:**
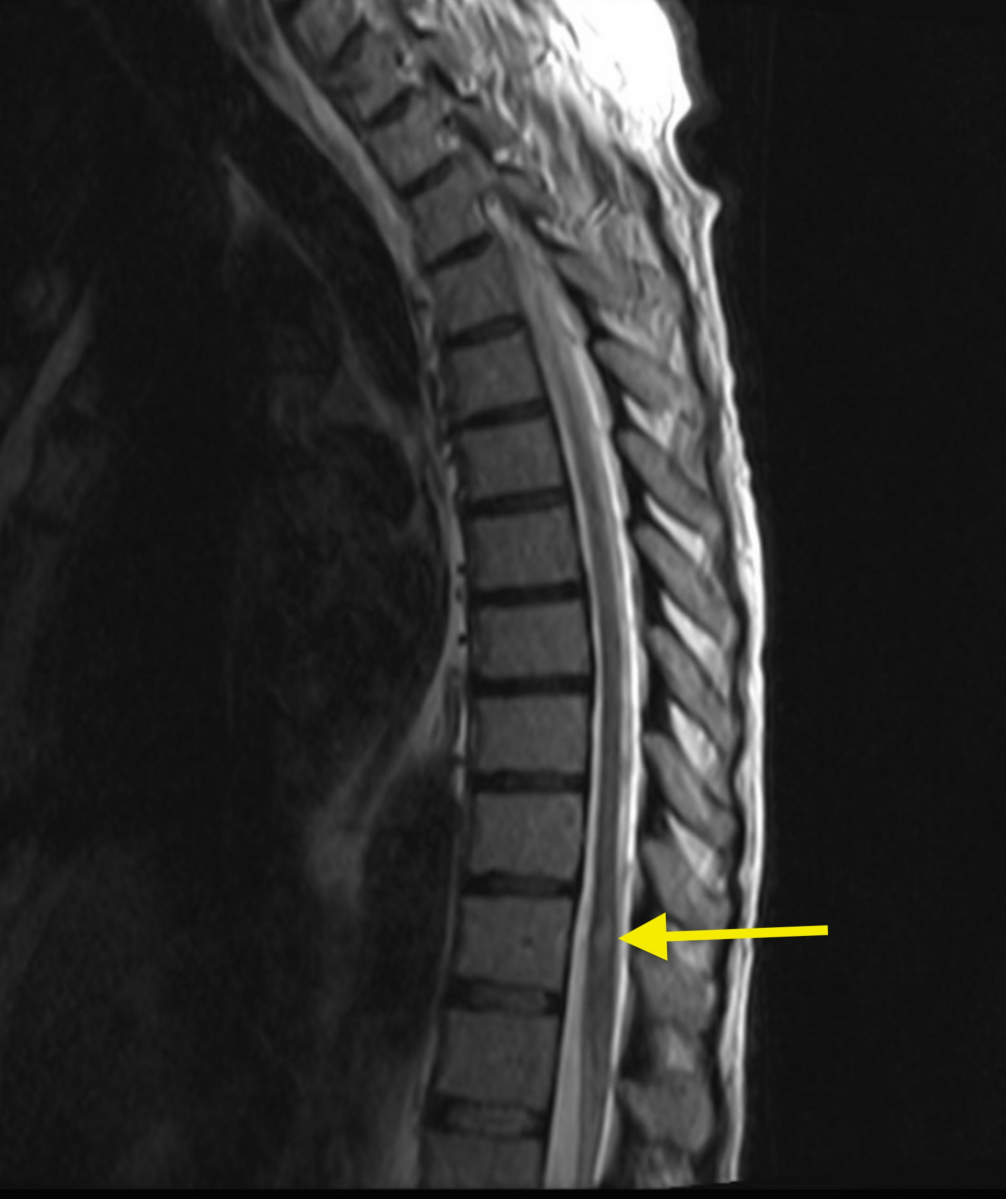
Magnetic resonance imaging (MRI) of the thoracic spine showing hyperintense lesion at T11 level (yellow arrow).

**Figure 7:**
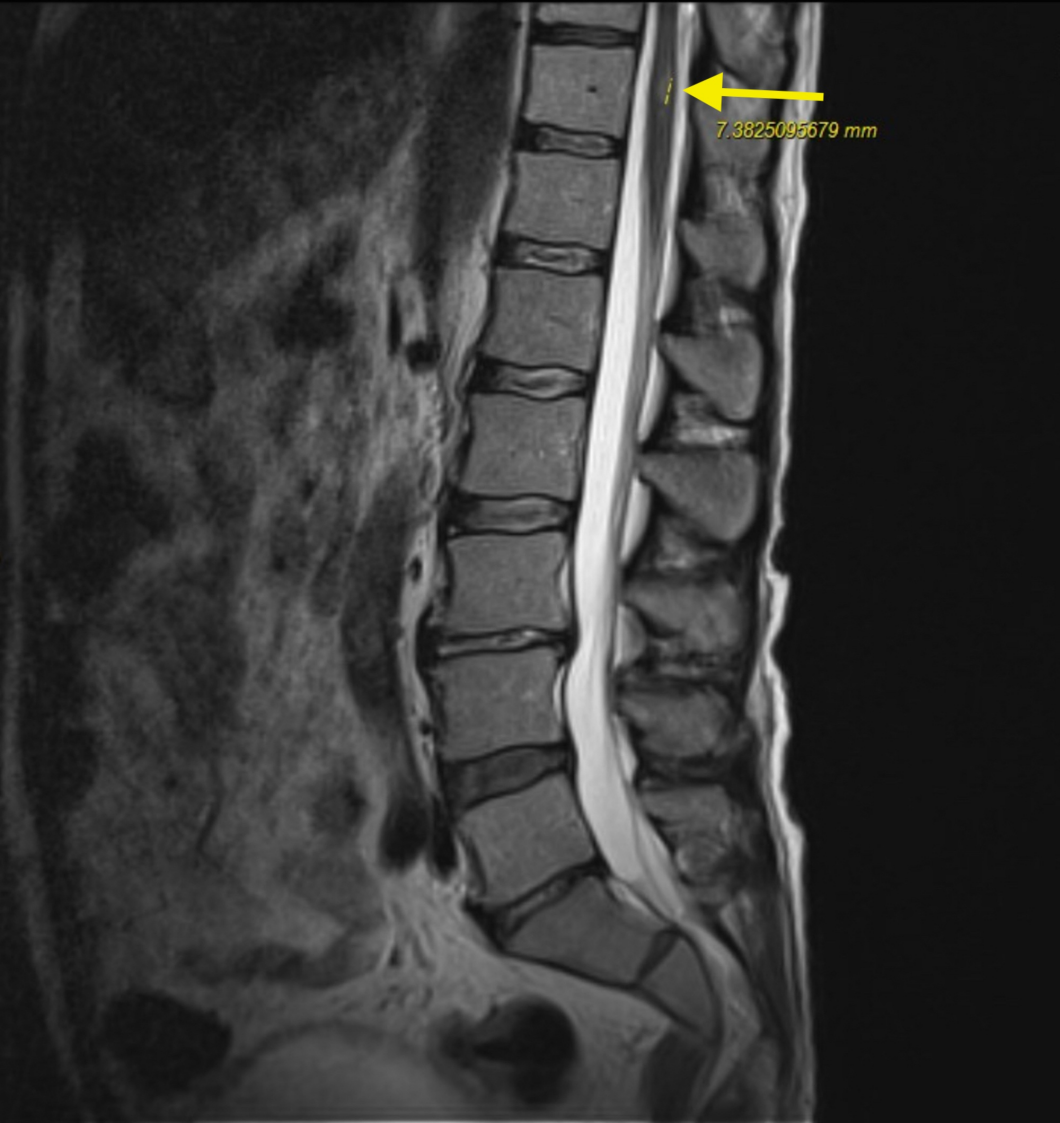
Magnetic resonance imaging (MRI) lumbar spine demonstrating 7 mm lesion and increased signal in superior margin of T11 (yellow arrow).

MRI of the cervical, thoracic and lumbar region also revealed numerous ring enhancing lesions (Figures 4-7).

**Figure 8:**
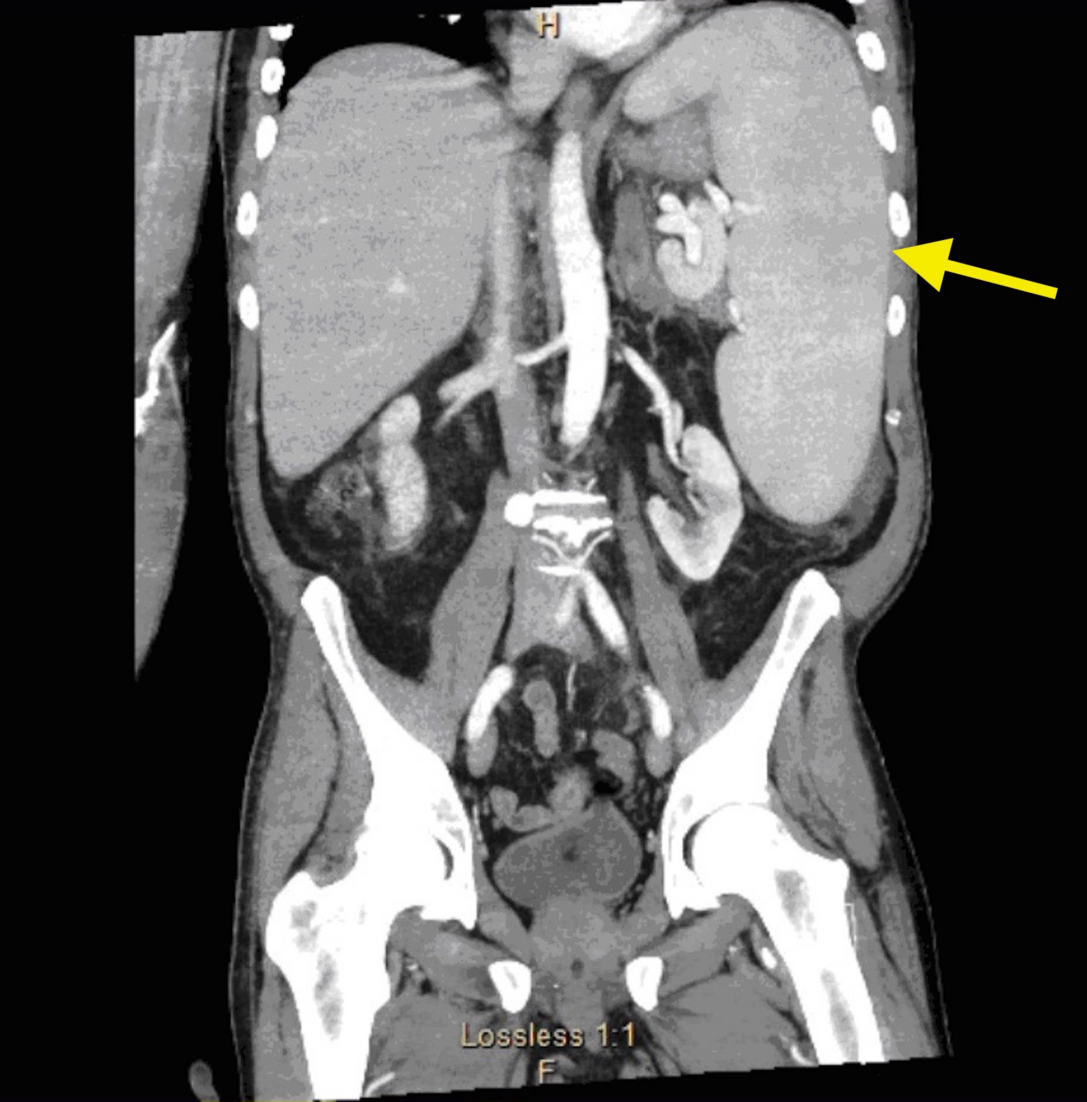
Computed tomography (CT) abdomen and pelvis demonstrating splenomegaly (yellow arrow).

The patient was started on Decadron and Keppra at this time. His antibiotic regimen was transitioned to liposomal amphotericin and meropenem. A CT chest, abdomen and pelvis was then ordered to search for any potential primary tumor. Results were significant for bilateral adrenal masses, 4.1 x 3 cm on the right, and 1.6 cm on the left, and splenomegaly (Figure 8).

Due to the patient’s poor mental status a continuous video electroencephalogram (EEG) was performed which revealed bilateral slowing consistent with encephalopathy and no epileptiform discharges or seizures. It was decided to next proceed with a lumbar puncture. LP revealed CSF glucose 28 mg/dL, protein 138 mg/dL, and white blood cell count (WBC) 43. Cryptococcal antigen is negative. Flow cytometry of the cerebrospinal fluid revealed no evidence of a B-cell or T-cell lymphoma. Antinuclear antibody (ANA) and hepatitis panel were also negative.

Due to LP findings consistent with low glucose levels the decision was made to start the patient on empiric antibiotics targeting fungal species. The patient was also started on high-dose steroids and maintained on this therapy with appropriate seizure prophylaxis.

The patient underwent right frontal stereotactic needle biopsy of right frontal lobe ring-enhancing lesion with the use of MRI-guided frameless stereotactic navigation. Brain biopsy pathology showed reactive astrocytosis, small narrow neck budding fungi consistent with Histoplasma species with secondary necrotizing vasculitis. Acid-fast bacilli stain performed was negative. Follow-up serology testing showed Histoplasma yeast 1:16, and Histoplasma mycelia 1:8. Gonorrhea and chlamydia polymerase chain reaction (PCR) were negative. Testing for rapid plasma reagin (RPR) and fluorescent treponemal antibody absorption (FTA-ABS), methicillin-resistant staphylococcus aureus (MRSA), and HIV were negative. CD4 count was 100.

His antibiotic regimen was switched to meropenem, amphotericin and itraconazole. However, the patient had deterioration in his neurological status becoming obtunded. He became minimally responsive to verbal stimuli and the family decided to proceed with hospice due to worsening clinical course and poor prognosis.

## Discussion

CNS involvement in histoplasmosis is a rare occurrence seen in a minority of patients with disseminated disease. Current literature suggests that 50-90% of immunocompetent individuals who become infected with Histoplasma remain asymptomatic [11-19]. Immunosuppression remains the greatest risk factor for progression of histoplasmosis to disseminated disease, increasing the risk by up to 10 times compared to immunocompetent individuals [4-6, 20]. Those patients at the greatest risk of developing disseminated disease include those with HIV, solid organ transplant, and hematologic malignancy, in addition to patients on tumor necrosis factor (TNF) inhibitors, corticosteroids or other cytotoxic drugs [20]. In addition to being at an increased risk for the development of disseminated histoplasmosis, patients with hematologic malignancy may present a unique diagnostic challenge to clinicians as their underlying infection may mimic malignancy. There have been a series of case reports documenting disseminated histoplasmosis being misdiagnosed as a primary hematologic malignancies [1-10]. Similarly, initial evaluation of our patient at this institution in Tennessee, was suspicious for a primary malignancy which may have delayed his diagnosis of histoplasmosis.

Disseminated histoplasmosis affects multiple organ systems [5,7]. These signs and symptoms vary depending on the patient’s immune status and the chronicity of the infection [15]. In the immunocompetent individual, an acute infection presents with fever, fatigue, weight loss, pancytopenia, and hepatosplenomegaly. If these patients have a chronic infection, the patient may experience hepatosplenomegaly, hepatic enzyme elevation, pancytopenia, gastrointestinal and/or oropharyngeal lesions. Additionally, adrenal involvement (for example: adrenal masses) is found in 80-90% of cases, however adrenal insufficiency is found in <10%. An immunocompromised individual may present with more severe symptoms, including respiratory distress, coagulopathy, renal and hepatic failure, and shock.

CNS histoplasmosis is one of the many organ systems affected by disseminated disease [6, 7]. It is found in 5-20% of patients and is more likely to occur in immunocompromised individuals. Additionally, these patients have a mortality rate of 20-40% and a relapse rate of 50%, so it is integral to correctly identify and diagnose patients in a timely fashion [14]. Patients may experience signs and symptoms associated with chronic meningitis, focal brain lesions, cerebral vasculitis with stroke syndrome including transient ischemic attack (TIA), encephalitis, and localized spinal cord involvement [11]. These may include headache, confusion, neck stiffness/rigidity, ataxia, weakness, visual complaints, altered mental status, focal deficit, and seizure. The patient may even eventually become obtunded.

The primary radiographic finding of CNS involvement is the presence of multiple ring-enhancing lesions within the CNS. Other diagnoses to consider in patients with ring-enhancing lesions include metastasis; bacterial abscesses including mycobacteria, nocardia, actinomyces, rhodococcus, and listeria; fungal abscesses including zygomycosis, coccidioides, aspergillus, and cryptococcus; parasitic abscesses including toxoplasmosis, neurocysticercosis, echinococcus, and entamoeba; glioblastoma; subacute infarct; contusion; inflammatory and demyelinating diseases including sarcoidosis, systemic lupus erythematosus (SLE), neuro-Behcet’s disease, Whipple’s disease, multiple sclerosis, and acute disseminated encephalomyelitis; radiation necrosis; and resolving hematoma.

Confirming a diagnosis of CNS histoplasmosis is commonly challenging. Low sensitivity of currently available tests, cross-reactivity with other fungal disease, and false positives in the setting of extra CNS Histoplasmosis attribute to these difficulties [9]. Therefore, a combination of multiple diagnostic modalities is required to make a diagnosis. Historically, the gold standard for confirming a diagnosis of CNS histoplasmosis has been CSF culture due to its excellent specificity and positive predictive value [9]. However, CSF culture has a low sensitivity for detecting Histoplasma and culture growth may take several weeks, potentially delaying diagnosis and treatment selection [9,16,17]. According to a recent multicenter retrospective study, the sensitivity for CNS culture was 19%, the specificity was 100%, and the positive predictive value was 100% [9]. Furthermore, the authors of this study found that CSF analysis for antigen and anti-Histoplasma antibodies using newer enzyme immunoassays yielded a sensitivity of 98% and specificity 90.8%, greatly improving the sensitivity over previously used assays. Nonetheless, there have been reported cases where CSF analysis was non-diagnostic and brain biopsy was required to identify the diagnosis [18]. In our patient, a biopsy was used to make the diagnosis as there was significant concern that his brain lesions were metastatic disease arising from his suspected adrenal mass.

The prognosis of CNS histoplasmosis varies depending on when the diagnosis is made and if the patient is immunocompromised or not. In general, the best outcomes are seen when the diagnosis is made earlier and treatment is started, along with the patient being immunocompetent. According to treatment guidelines, when a patient is diagnosed with CNS histoplasmosis, he/she should be initially started on AMB-L (liposomal amphotericin B) for four to six weeks followed by itraconazole for at least one year [1-20]. This is secondary to evidence showing decreased mortality, reduced toxicity, superior pharmacodynamic characteristics compared to other treatment options, and greater concentration in brain tissue. With this treatment, a multicenter retrospective study showed the overall one year survival rate was 74% [6]. Additionally, there is a 6% recurrence rate. The survival rates were much higher than previously studied and the recurrence rate much lower. One theory is that after Infectious Diseases Society of America (IDSA) guidelines indicated AMB-L should be first-line treatment, patients were receiving the optimal therapy for survival. Additionally, over half of these patients had normal functional statuses at the last follow-up. However, 14% (primarily immunocompromised patients) had severely impaired functional status. Overall, as early recognition of the disease has risen, diagnostic studies have improved, and treatments have become more specific and effective, the prognosis of patients has vastly improved.

## Conclusions

CNS manifestations of histoplasmosis occur in a minority of patients with disseminated disease. These findings are often non-specific and variable from one patient to another, making the clinical diagnosis of CNS histoplasmosis difficult for clinicians. Furthermore, some patients with CNS histoplasmosis may present without pulmonary disease or clear signs of infection. Due to the difficulty of recognizing CNS histoplasmosis purely from clinical presentation, clinicians are tasked with obtaining a thorough history and physical to identify certain risk factors that have traditionally placed individuals at increased risk of contracting disseminated histoplasmosis.
